# Re-Examination of PGT-A Detected Genetic Pathology in Compartments of Human Blastocysts: A Series of 23 Cases

**DOI:** 10.3390/jcm13113289

**Published:** 2024-06-03

**Authors:** Andrei V. Tikhonov, Mikhail I. Krapivin, Olga V. Malysheva, Evgeniia M. Komarova, Arina V. Golubeva, Olga A. Efimova, Anna A. Pendina

**Affiliations:** D.O. Ott Research Institute of Obstetrics, Gynecology and Reproductology, Mendeleevskaya Line 3, 199034 Saint Petersburg, Russia

**Keywords:** preimplantation genetic testing, blastocyst, aneuploidy, trisomy, monosomy, trophectoderm, inner cell mass, assisted reproductive technologies

## Abstract

**Background:** In recent years, preimplantation genetic testing for aneuploidies (PGT-A) has become widespread in assisted reproduction. However, contrary to expectations, PGT-A does not significantly improve the clinical outcomes of assisted reproductive technologies. One of the underlying reasons is the discordance between the PGT-A results and the true chromosomal constitution of the blastocyst. In this case series, we re-examined the PGT-A results in trophectoderm (TE) re-biopsies and in the two isolated blastocyst compartments—the TE and the inner cell mass (ICM). **Methods:** This study enrolled 23 human blastocysts from 17 couples who were referred for assisted reproduction. The blastocysts were unsuitable for uterine transfer due to the chromosomal imbalance revealed by PGT-A using array comparative genomic hybridization (aCGH) (n = 11) or next-generation sequencing (NGS) (n = 12). The re-examination of the PGT results involved two steps: (1) a TE re-biopsy with subsequent aCGH and (2) blastocyst separation into the TE and the ICM with a subsequent cell-by-cell analysis of each isolated compartment by fluorescence in situ hybridization (FISH) with the DNA probes to chromosomes 13, 16, 18, 21, and 22 as well as to the PGT-A detected imbalanced chromosomes. **Results:** In 8 out of 23 cases, the PGT-A results were concordant with both the re-biopsy and the isolated TE and ICM analyses. The latter included the diagnoses of full non-mosaic aneuploidies (five cases of trisomies and two cases of monosomies). In one case, the results of PGT-A, aCGH on the TE re-biopsy, and FISH on the isolated TE showed Xp tetrasomy, which contrasted with the FISH results on the isolated ICM, where this chromosomal pathology was not detected. This case was classified as a confined mosaicism. In 4 out of 23 cases, the results were partially discordant. The latter included one case of trisomy 12, which was detected as non-mosaic by PGT-A and the re-biopsy and as mosaic by FISH on the isolated TE and ICM. This case was classified as a true mosaicism with a false negative PGT-A result. In 11 out of 23 cases, the re-examination results were not concordant with the PGT-A results. In one of these discordant cases, non-mosaic tetraploidy was detected by FISH in the isolated TE and ICM, whereas the PGT-A and the TE re-biopsy failed to detect any abnormality, which advocated for their false negative result. In two cases, the re-examination did not confirm full aneuploidies. In eight cases, full or partial mosaic aneuploidies as well as chaotic mosacism were not confirmed in the isolated TE nor the isolated ICM. Thus, in 47.8% of cases, the PGT-A results did not reflect the true chromosomal constitution of a blastocyst. **Conclusions:** The PGT results may have different prognostic value in the characterization of the chromosomal constitution of a blastocyst. The detected non-mosaic aneuploidies have the highest prognostic value. In stark contrast, most PGT-identified mosaic aneuploidies fail to characterize the true chromosomal constitution of a blastocyst. Once detected, a differential diagnosis is needed.

## 1. Introduction

Assisted reproductive technologies (ARTs) is the area of medicine that is undergoing many dynamic advances. New emerging approaches, as well as the ongoing development of existing ART techniques, allows for fertility issues to be resolved in a growing number of couples.

Preimplantation genetic testing (PGT) is an unarguable breakthrough in ART allowing for the evaluation of the genetic status of an embryo prior to transfer. Since its first successful implementation, the PGT procedure has seen rapid development. Currently, PGT refers to a broad range of techniques, including fluorescence in situ hybridization (FISH) and comprehensive molecular methodologies, such as array comparative genomic hybridization (aCGH), quantitative polymerase chain reaction (qPCR), single-nucleotide polymorphism (SNP) array, and next-generation sequencing (NGS). Alongside benefits, each type of testing has limitations that inevitably contribute to a certain subset of diagnostic errors [1,2,3,4,5,6]. Nevertheless, the evidence of the embryo genetic constitution obtained using these methods is decisive for uterine transfer.

Lately, preimplantation genetic testing for aneuploidies (PGT-A) has become a widely available and routinely performed procedure. Despite our expectations, however, there is little improvement in ART clinical outcomes, giving rise to assumptions that PGT results might be discordant with the true chromosomal constitution of a blastocyst. This is supported by the evidence of 100 uterine transfers, when blastocysts with PGT-A detected mosaic aneuploidies that eventually produced healthy offspring [7,8,9,10,11].

The discordance between the PGT-A results for a few blastomeres or trophectoderm (TE) cells and the true chromosomal constitution of a blastocyst provokes controversies on the method of choice, considering laboratory manipulations and techniques, as well as the rationale of testing in general [12,13]. Indeed, on the one hand, PGT is able to diagnose true genetic imbalances and does not allow for abnormal embryos to be unknowingly transferred. On the other hand, potential false positive results reduce transfer, pregnancy, and childbirth chances [14,15,16]. Moreover, there is still a certain risk of PGT-A delivering false negative results, which is supported by few cases of pregnancy failure or early abortion following the uterine transfer of genetically balanced embryos [17]. Due to assumed stage-specific chromosomal heterogeneity, the preimplantation development of a human embryo itself seems to be another source of false results by PGT-A considering the embryo’s self-correction ability through the clonal depletion of aneuploid cells and the propagation of karyotypically normal cells [18,19,20,21].

Currently, studies are attempting to understand the concordance rate of PGT-A results with the true chromosomal constitution of a blastocyst and whether a trophectoderm (TE) re-biopsy is necessary to improve the validity of PGT-A [2,15,22,23,24,25,26]. Nevertheless, the results are contradictory, and there is still a paucity of clear-cut indications for PGT-A and insufficient evidence of its benefits, so it is difficult to select the most optimal screening strategy [14,15,23,26]. Therefore, the objective of our research is to re-examine the PGT-A results for both blastocyst compartments—the TE and the inner cell mass (ICM).

## 2. Materials and Methods

### 2.1. Patients and Samples

The present study enrolled a total of 23 human blastocysts obtained from 17 couples (the mean age of the female partners was 37.72 ± 5.81 years) who were referred to the D.O. Ott Research Institute of Obstetrics, Gynecology and Reproductology (St. Petersburg, Russia) for in vitro fertilization (IVF) and PGT-A. A controlled ovarian stimulation was performed using a recombinant and/or urinary gonadotropin antagonist protocol, which was described in our earlier study [27]. Oocyte retrieval and fertilization by IVF or intracytoplasmic sperm injection (ICSI) was performed in our research center according to the standard clinical practice [28]. Embryos were cultured in G-TL medium (Vitrolife, Gothenburg, Sweden). PGT-A by aCGH (n = 11) or NGS (n = 12) was performed on day 5 of TE biopsies. The data on the PGT-A techniques and results for the analyzed embryos are summarized in Table 1. To conduct aCGH, Agilent Cytogenomics V3.0 (Santa Clara, CA, USA) was used with a resolution of 5 mbp. NGS was performed using Illumina MiSeq and BluFuse Multi v4.3 (San Diego, CA, USA) with a resolution of 5 mbp. After the TE biopsy, all embryos were immediately cryopreserved using Kitazato vitrification media (Vitrification Media, Kitazato, Japan).

### 2.2. Re-Examining the PGT-A Results in the TE Re-Biopsies and the Genetic Analysis of the Isolated TE and ICM

**Figure 1:**
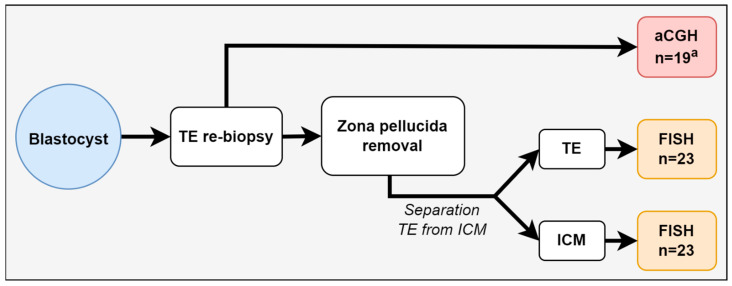
Study design. ^a^ In 4 out of 23 re-biopsy cases, no whole genome amplification product was obtained, making further aCGH analysis impossible.

The re-examination of the PGT-A results included two steps (Figure 1). At the first step, the TE re-biopsy was performed. For this purpose, cryopreserved blastocysts were thawed using Kitazato thawing media (Thawing Media, Kitazato, Japan) according to the manufacturer’s protocol and placed in the G-TL culture medium (10145, Vitrolife, Sweden) for 12–16 h (5% O_2_, 6% CO_2_). The Gardner grading system was used for the blastocyst assessment [29]. The blastocysts included in the study were of 3BB quality or higher. The TE re-biopsy was performed using the OCTAX laser. Each re-biopsy procedure produced a total of 4–9 TE cells. The aCGH analysis of the TE re-biopsies was performed using Agilent Cytogenomics V3.0 (Santa Clara, CA, USA) according to the standard protocol used in our laboratory [30]. In 4 out of 23 re-biopsy cases, no whole genome amplification (WGA) product was obtained, making a further aCGH analysis impossible. Therefore, aCGH was performed in 19 out of the total 23 TE re-biopsies.

At the second step, following the TE re-biopsy, zona pellucida was removed, and the Octax laser (Vitrolife, Sweden) was applied to each zona-free blastocyst to separate the TE and the ICM. Then, the TE and ICM cells were fixed on glass slides for further cytogenetic preparations, which were conducted according to the regularly applied laboratory protocol for paucicellular samples [31,32,33,34] with minor modifications. Then, the TE and ICM preparations for each blastocyst were subject to fluorescence in situ hybridization (FISH) using the Vysis MultiVysion PB Multi-Color FISH Probe Kit (Abbott Molecular) for chromosomes 13 (13q14.2), 16 (16q11.2 Satellite II), 18 (18p11.1–q11.1 Alpha Satellite), 21 (21q22.13–q22.2), and 22 (22q11.2) and abnormal chromosomes that were identified earlier at the initial PGT-A. In each blastocyst, 3 to 46 cells were available for FISH analysis. Microscopic analysis was performed using a Leica DM2500 microscope equipped with a high-resolution Leica DFC345 FX camera and Leica Application Suite V3 software.

The rate of chromosomally imbalanced blastocysts (with mosaic and non-mosaic aneuploidies) was used to calculate the false positive or false negative result probability for the total number of analyzed blastocysts. The false positive and false negative result probabilities were compared using Fisher’s exact test.

### 2.3. Ethical Approval

This study was conducted in accordance with the Declaration of Helsinki and approved by the Ethics Committee of the D.O. Ott Research Institute of Obstetrics, Gynecology and Reproductology (protocol no. 120, 21 July 2022). All patients signed an informed consent form.

## 3. Results

### 3.1. Comparing PGT-A and Trophectoderm Re-Biopsy Results

In 19 out of 23 blastocysts, an aCGH was performed on the TE re-biopsy. Four out of twenty-three blastocysts failed the WGA, rendering further investigation impossible. The TE re-biopsy evidence of genetic imbalance was concordant with the PGT-A results in nine (47.4%) cases (cases 1–8 and 10 in Table 1) of a total of 19 blastocysts. The nine concordant blastocysts included five blastocysts with the PGT-A performed by aCGH and four blastocysts performed by NGS (Table 1). The re-biopsy confirmed genetic imbalance in eight full aneuploidy cases—including six trisomy cases (trisomy 19 in two cases (cases 1 and 5) and trisomy 22, 16, 13, and 12 in four cases (cases 2, 6, 7, 10))—and two monosomy cases (monosomy 18 (case 3) and double monosomy 4 and 21 (case 8)). In addition, a single case of partial tetrasomy of the short arm of chromosome X (case 4) was registered.

Three cases (15.8%) revealed partially concordant PGT-A and re-biopsy results, with the PGT-A performed by aCGH in one case and by NGS in two cases. In one case, incomplete concordance was produced by the re-biopsy failure to confirm the partial monosomy of the short arm of chromosome 2 (case 9); another discordant case revealed multiple mosaic aneuploidies detected by the re-biopsy (case 11); the third case showed mosaic trisomy, which was detected at the re-biopsy as well (case 12).

In seven cases (36.8%), however, the PGT-A results were totally discordant with the re-biopsy evidence. Each of the samples revealed mosaic aneuploidies detected by PGT-A only without re-biopsy evidence or, in contrast, by re-biopsy only without any supporting PGT-A evidence.

### 3.2. Re-Examination of PGT-A and TE Re-Biopsy Detected Genetic Imbalances by FISH in Isolated TE and ICM

**Figure 2:**
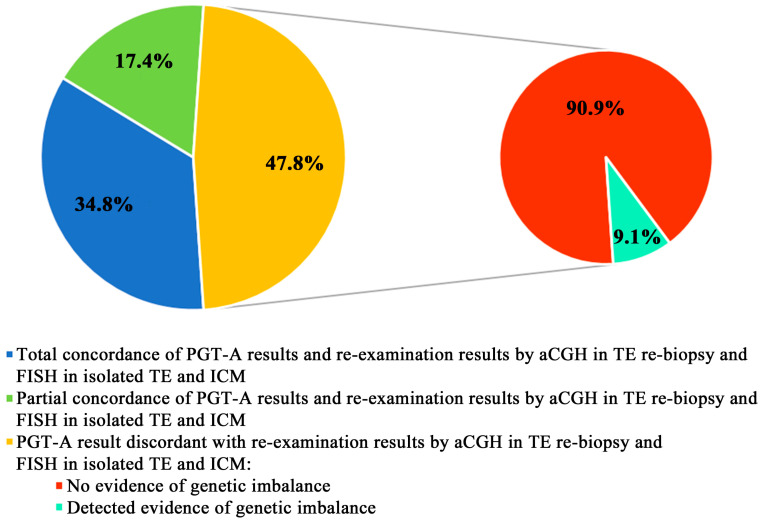
The correspondence of the PGT-A results and the re-examination results by aCGH in the TE re-biopsy and FISH in the isolated TE and ICM.

In 8 out of 23 cases (34.8%), PGT-A and the re-biopsy revealed genetic imbalances that were confirmed by FISH in the TE and ICM (Figure 2). In seven cases, the FISH results for the TE and ICM showed total concordance with the PGT-A and TE re-biopsy results (see cases 1 to 3 and 5 to 8 in Table 1), revealing full aneuploidies—five full trisomy cases (two trisomy 19 cases and three trisomy 22, 16, and 13 cases (Figure 2)) and two full monosomy cases (monosomy 18 and double monosomy 4 and 21). In case 4, PGT-A and the TE re-biopsy revealed the Xp tetrasomy, which was confirmed in the isolated TE but went undetected in the isolated ICM. Therefore, we classified it as the inter-tissue mosaicism.

In four cases (17.4%), the FISH results in the isolated TE and ICM were discordant with the PGT-A and TE re-biopsy results (Figure 2). One embryo classified as trisomic for chromosome 12 by PGT-A and the TE re-biopsy showed mosaicism for trisomy 12 in the isolated TE and ICM (case 10). This case was classified as true mosaicism with a false negative PGT-A for mosaic trisomy detection. Notably, however, the trisomic clone in both blastocyst compartments constituted some 50%: trisomy 12 was detected in 19 of 29 TE cells and in 17 of 35 ICM cells. Two embryos with confirmed full trisomy 22 in both compartments eventually produced non-concordant re-examination results due to failure to verify partial monosomy 2 (case 9) and multiple mosaic full trisomies (case 11) in both the TE cells and the ICM. Mosaic trisomy 8 detected by the re-biopsy with non-mosaic trisomy 8 detected by both the initial PGT-A and then by FISH in the isolated ICM and TE cells was the other cause of non-concordant findings (case 12).

In 11 (47.8%) out of 23 cases, PGT-A, the TE re-biopsy, and re-examination by FISH in isolated TE cells and the ICM showed totally discordant findings (Figure 2). In one of the eleven embryos (case 14), the isolated TE and ICM presented with total tetraploidy that was not detected by PGT-A nor the re-biopsy, with PGT-A producing evidence of partial mosaic monosomy for chromosome 2 and no evidence of imbalance being detected by the TE re-biopsy. The PGT-A result for this case can be classified as false negative for tetraploidy and false positive for partial chromosome 2 monosomy. The 11 cases also included two blastocysts with no evidence of total aneuploidies following re-examination (non-confirmed full trisomy 8 in case 23 and full monosomy 4 in case 19). In eight cases (34.8%), both the ICM and TE re-examination failed to confirm total or partial mosaic aneuploidies (cases 13, 15, 16, 18, and 20 to 22) and chaotic mosaicism (case 17). The above PGT-A results can be considered false positive.

### 3.3. Discordant PGT-A Findings and Probability Assessment of False Positive or False Negative Results

Based on their PGT-A findings, the blastocysts (n = 23) were split into two groups to assess the probability of false positive or false negative results: non-mosaic aneuploidies (n = 14) and mosaic aneuploidies (n = 9). The PGT-A methods showed a 21.43% chance of false positive non-mosaic aneuploidy (trisomies and monosomies) results (3 of 14). None of the nine PGT-A-detected mosaic aneuploidies were confirmed. The PGT-A-detected mosaic aneuploidies had a significantly higher risk of obtaining a false positive result than the non-mosaic aneuploidies (Fisher’s exact test, *p* = 0.0003). The non-mosaic and mosaic aneuploidies yielded probabilities of false negatives of 11.1% (1 of 9) and 7.1% (1 of 14), respectively, showing no significant difference in the probability rates (Fisher’s exact test, *p* = 1.00). Overall, on the one hand, PGT-A has poor predictive value for a mosaic aneuploid diagnosis and a high predictive value in detecting non-mosaic aneuploidies on the other hand.

## 4. Discussion

Accurately characterizing the chromosomal constitution of blastocysts is critical since chromosomally balanced embryos largely contribute to ART success [35,36,37,38]. The necessity of PGT-A itself is beyond doubt considering the high incidence of abnormal chromosome segregation in meiosis during gametogenesis and in mitosis during cleavage divisions [17,36,37,39,40,41,42,43]. Meiotic errors are usually less common in spermatogenesis and mostly arise in oogenesis, increasing significantly in frequency with maternal age. Thus, evidence suggests that the majority of aneuploidies (90.1%) arise in meiosis I (69.4%) of oogenesis, with less arising in meiosis II (30.6%). In contrast, spermatogenesis shows an incidence rate of aneuploidies under 10% [44]. However, whatever the age, the risk of aneuploid gametes arising from meiotic errors is never zero, whereas chromosomal rearrangements and mitotic nondisjunction are not age-dependent [44,45,46]. This is the reason why the utility of PGT-A is not limited by an advanced maternal reproductive age. PGT-A significantly reduces the risk of the uterine transfer of chromosomally abnormal embryos, thus diminishing the risk of ART failure, miscarriage, or birth with chromosomal abnormalities [17,47].

However, whether the PGT-A results obtained with cutting-edge techniques and procedures characterize the true chromosomal constitution of a blastocyst is still an unresolved issue. This can be explained by truly biological reasons, such as the self-correction of chromosomally abnormal embryos, embryo ‘normalization’ to a euploid state [19,20,21,48], and true inter-tissue and tissue-limited mosaicism [20,49,50], as well as by a biopsy or PGT-A procedural specificity [51,52]. In recent years, investigators have become increasingly aware of this problem, which has given rise to abundant research papers [15,24,25,26,53,54]. In summary, the findings reported by fellow investigators [15,24,46,55] allow for the PGT-A verification results to be split into true, false positive, and false negative results. True results refer to total concordance between the PGT-A results and the true chromosomal constitution, occurring in 34.8% in our study and varying from 33% to 84.3% in other studies [2,14,15,23,24,25,26,53]. As a rule, all studies, as well as the present paper, report the total concordance in non-mosaic aneuploidies of meiotic origin and in unbalanced chromosomal rearrangements inherited from one of the parents [23,25]. Thus, chromosomal abnormalities of meiotic origin, affecting all cells of an embryo, demonstrate the highest detection rate by PGT-A.

We identified false negative PGT-A results in two cases characterized by true mosaicism and tetraploidy. A true mosaic case had a combination of trisomy 12 and disomy 12 clones in the ICM and TE. Trisomy 12 clones constituted 65.5% in TE cells and 48.6% in the ICM. However, neither the primary TE biopsy carried out using NGS nor the subsequent aCGH re-biopsy revealed mosaicism, suggesting that the ability of the molecular WGA technique to detect true mosaicism is still not deprived of bias. The other false negative case was a tetraploidy overlooked by both primary and aCGH repeat biopsies. It is noteworthy, however, that tetraploidy is found in approximately 3% of human miscarriages [56]. False negative PGT-A results are of particular concern since the uterine transfer of such false euploid embryos is associated with adverse ART outcomes. Such cases suggest extra evidence that an invasive prenatal diagnosis is required to exclude fetal chromosomal abnormalities in pregnancies that follow the uterine transfer of a PGT-identified euploid embryo and exhibit markers of chromosomal abnormality.

Almost half of the false positive PGT-A results revealed in this study were characteristic of mosaic aneuploidies, including chaotic mosaicism. Verification has not confirmed any such PGT-A detected mosaic aneuploidies. Similar results were reported by fellow investigators, suggesting that mosaic aneuploidies detected using a primary TE biopsy are associated with extremely poor forecasting value in revealing the true chromosomal constitution of a blastocyst [22,26,57]. In addition, according to our findings, verification did not confirm mosaic aneuploidies identified using re-biopsy. A possible explanation may refer to some biological reasons, such as the fact that mosaicism is limited to biopsy cells only, as well as to technical constraints, such as WGA artifacts, etc. Unfortunately, the PGT-A-identified mosaic aneuploidies do not allow for biological and technical limitations to be discerned and make it impossible to reveal the true cause of mosaicism. In the case of technical interference, with the embryo chromosomally balanced, uterine transfer can produce healthy pregnancy and childbirth. Recent investigations have reported numerous cases of healthy pregnancies and live births from embryos deemed mosaic after PGT-A [7,8,9,10,11,36,58]. Healthy childbirths were observed after transferring embryos with PGT-A-detected monosomies 2 and 5 and double monosomies 5 and 7, 4 and 10, and 6 and 15 [8]. The underlying reasons for false positive monosomies may include diagnostic technical issues or embryo genome self-correction. It is common knowledge that aneuploidies hinder cell proliferation and embryo viability [59]. Studies in chimeric mouse embryos have shown that in stark contrast to euploid cells, aneuploid TE cells have slow DNA replication, whereas those in the ICM are eliminated by apoptosis [60]. In cases of low to middle rates of aneuploidy, the presence of sufficient euploid cells can rescue embryo development. Nevertheless, high aneuploidy rates exceeding the threshold values invariably result in embryo death [60]. A few human blastocysts originally diagnosed as mosaic and cultured in vitro remained viable, showing a complete loss of aneuploid cells. However, high fractions of aneuploid cells were strongly associated with blastocyst death [61]. Thus, high rates of abnormal cells and/or complex aneuploidies involving more than one chromosome can significantly impede embryo survival. Embryos with a low fraction of genetically abnormal cells and/or segmental aneuploidies have greater viability potential. This fact sheds light on the discrepancies in implantation and birth rates following mosaic embryo transfers [11]. A retrospective study by Capalbo et al. did not show a significant impact on birthrates following the uterine transfer of PGT-A-confirmed mosaic embryos, with healthy offspring produced from 42.9% of low-grade mosaic embryos, 42.0% of medium-grade mosaic embryos, and 43.4% of euploid embryos [10]. In contrast, the study by Viotti et al. reported 37.0% and 52.3% of developing pregnancies/births following the transfer of PGT-A-diagnosed mosaic and euploid embryos, respectively [11]. In fact, a lower incidence of successful implantations and higher rates of miscarriages following the transfer of mosaic embryos has been reported by many fellow investigators [11,17,25,36,62,63,64]. Moreover, a pregnancy and live birth with chromosomal defects produced by a true mosaic blastocyst transfer should be taken into consideration.

Notably, our study shows that out of 23 cases with PGT-A-identified abnormality, 11 cases were not confirmed by verification, with the blastocyst revealing a normal chromosomal constitution. It is highly likely that the uterine transfer of these embryos would have resulted in a favorable ART outcome. Fellow investigators have reported similar scenarios. Thus, following PGT-A verification, three studies reported that normal chromosomal constitution of the blastocyst was detected in 9 out of 22 cases (40.9%) [2], in 5 out of 100 cases (5%) [25], and in 18 out of 26 cases (69.2%) [26], respectively. Thus, mosaicism poses a critical diagnostic problem, whereas detected mosaic aneuploidies reveal an extremely low prognostic value. Munne et al. [3] suggested the following classifications for PGT-A results: 1. euploid, with the highest implantation potential; 2. mosaic, with an elevated miscarriage rate and reduced (but not null) implantation potential; and 3. aneuploid, with a very low probability of implantation, a significantly elevated risk of miscarriage, a probably close to zero potential for a live birth, and significant fetal and neonatal risk. PGDIS recommends that clinical laboratories should refrain from classifying a mosaic embryo as being unsuitable for transfer as this may restrict subsequent treatment options [65]. So far, the current PGT-A techniques fail to yield sufficient evidence allowing for true mosaicism and technical errors to be differentiated. Meanwhile, re-biopsy is also susceptible to errors, showing little significance for the detection of true mosaic aneuploidies. Capablo et al. showed that high-grade mosaicism (50–70%) in a single TE sample was commonly associated with non-mosaic aneuploidy throughout the embryo (including the ICM) in 65% of cases [10]. The above data allow us to conclude that each preimplantation embryo diagnosed as mosaic requires a more careful and personalized evaluation considering the diagnostic technique, the affected chromosomes, and the mosaicism rate. Therefore, a PGT-A-detected mosaic embryo should never be considered euploid. In the case that a decision is made to transfer such an embryo into the uterus, the pregnancy shall be managed with ultimate caution. The latter justifies the dire need for additional differential diagnosis techniques based on entirely innovative approaches, such as differential gene expression in mosaic blastocysts [66]. These technologies may suggest innovative diagnostic criteria allowing for the mosaic chromosomal constitution of a blastocyst to be verified.

The present study has underlying advantages and limitations. To re-examine the PGT-A results, this study used two techniques: aCGH and FISH. aCGH on a TE re-biopsy enabled the verification of the initial PGT-A results, which were also obtained utilizing a DNA analysis by molecular genetic techniques—aCGH or NGS. FISH, in turn, enabled a manual analysis at a single-cell level for both blastocyst compartments and allowed us to identify potential biological causes (confined mosaicism) of discordance between the PGT-A results and clinical outcomes. The FISH method does not require WGA, thus eliminating WGA-associated risks of technical errors, including errors due to cell damage during sampling. However, FISH is restricted to the evaluation of individual chromosome loci without the possibility of assessing the whole genome. Therefore, the present study mainly utilized FISH with DNA probes to the loci of interest to verify the initial PGT-A results based on aCGH or NGS, or aCGH for TE re-biopsies. The FISH method is notably the gold standard of mosaicism diagnosis, allowing experts to ‘map’ the genetic material of cells and identify the exact number of cells with and without chromosomal pathology, even for low-grade mosaicism [67,68]. The sample size in our study is limited, considering that the analyzed human blastocysts were initially obtained for the ART and were often not readily available for experimental research. In addition, for ethical reasons, we had no access to euploid PGT-A blastocysts to establish a control group. On the one hand, such limitations prevent investigators from making overgeneralized conclusions, especially regarding the incidence of false positive and false negative PGT-A results. On the other hand, each embryo in the ART procedure is considered individually with regard to the PGT-A results and the decision to transfer the embryo following preliminary medical and genetic counseling. This allows us to assume that the presented findings are relevant and valuable for clinical practice.

In conclusion, different PGT-A results are distinct in terms of their prognostic value for blastocyst chromosomal constitution characterization. The identified non-mosaic aneuploidies show the highest prognostic value. If detected following PGT-A, such aneuploidies are highly likely to reflect the true chromosomal constitution of a blastocyst. On the contrary, in most cases, PGT-identified mosaic aneuploidies fail to characterize the true chromosomal constitution of a blastocyst. Once detected, additional differential diagnosis based on innovative approaches is critically required.

## Figures and Tables

**Table 1:** The PGT-A and re-examination results in the TE re-biopsies and in the isolated TE and ICM. ^a^ PB—Vysis MultiVysion PB Multi-Color FISH Probe Kit: 13 (13q14.2), 16 (16q11.2 Satellite II), 18 (18p11.1–q11.1 Alpha Satellite), 21 (21q22.13–q22.2), and 22 (22q11.2). ^b^ PB-N—unless otherwise specified, FISH with Vysis MultiVysion PB revealed two copies of chromosomes.

№	Patient’s Age, Years	Blastocyst Quality, Gardner Grade	PGT-A Technique	PGT-A Result	Results of PGT-A Re-Examination			Concordance of PGT-A Results and Their Re-Examination
					by aCGH in TE Re-Biopsy	by FISH in Isolated		
						TE [Cell Number]	ICM [Cell Number]	
1	38	4AA	aCGH	arr(19)x3	arr(19)x3	PB ^a^-N ^b^, 19x3 [12]	PB-N, 19x3 [15]	full
2	38	4AA	aCGH	arr(22)x3	arr(22)x3	PB-N, 22x3 [43]	PB-N, 22x3 [14]	full
3	43	3BB	aCGH	arr(18)x1	arr(18)x1	PB-N, 18x1 [4]	PB-N, 18x1 [20]	full
4	34	2AA	aCGH	arr(Xp)x4	arr(Xp)x4	PB-N, CEPXx4, Xp22.3x4 [10]	PB-N, CEPXx2 [7]	full
5	38	3BB	aCGH	arr(19)x3	arr(19)x3	PB-N, 19x3 [4]	PB-N, 19x3 [2]	full
6	33	4AA	NGS	seq(16)x3	arr(16)x3	PB-N, 16x3 [8]	PB-N, 16x3 [8]	full
7	33	5AB	NGS	seq(13)x3	arr(13)x3	PB-N, 13x3 [46]	PB-N, 13x3 [35]	full
8	42	4AA	NGS	seq(4)x1, seq(21)x1	arr(4,21)x1	PB-N, 4p11-q11x1, Tel4qx1, 21x1 [31]	PB-N, 4p11-q11x1, Tel4qx1, 21x1 [23]	full
9	34	3BB	aCGH	arr(2p25.3p21)x1, (22)x3	arr(22)x3	PB-N, 2p24x2, 2p11.1-q11.1x2, 11p11.11-q11x2, 22x3 [5]	PB-N, 2p24x2, 2p11.1-q11.1x2, 11p11.11-q11x2, 22x3 [5]	partial
10	39	5AB	NGS	seq(12)x3	arr(12)x3	PB-N, 12x3[19/29]/12x2 [10/29]	PB-N, 12x3[17/35]/12x2 [18/35]	partial
11	45	5AB	NGS	seq(22)x3	arr(1,5,7,20)x2~3, (22)x3	PB-N, 1x2, 5x2, 7x2, 20x2, 22x3 [8]	PB-N, 1x2, 5x2, 7x2, 20x2, 22x3 [12]	partial
12	28	5BB	NGS	seq(8)x3	arr(8)x2~3	PB-N, 8q24.21x3 [12]	PB-N, 8q24.21x3 [8]	partial
13	29	3BB	aCGH	arr(6,9,11,13)x2~3	arr(1-22,X)x2	PB-N, CEP6x2, CEP7x2, CEP9x2, CEP11x2, CEPXx2 [4]	PB-N, CEP6x2, CEP7x2, CEP9x2, CEP11x2, CEPXx2 [7]	no
14	31	5BB	aCGH	arr(2q32.1 q27.3)x1[0,3]	arr(1-22,X)x2	PBx4, CEPXx4 [2]	PBx4, CEPXx4 [12]	no
15	50	5AB	aCGH	arr(2p12p13)x1, (7p11.2p12)x1, (18)x1~2, (22q12.3q13)x1	arr(1-22,X)x2	PB-N, 22q13.3x2 [5]	PB-N, 22q13.3x2 [15]	no
16	38	4AA	aCGH	arr(22)x2~3	arr(1-22)x2	PB-N [31]	PB-N [17]	no
17	36	4BB	aCGH	chaotic mosaicism	arr(XY)x1, (1-22)x2	PB-N [4]	PB-N [7]	no
18	36	6AA	NGS	seq(16)x3 [0.2]	arr(Xq22.3q28)x1, (X)x2	PB-N, CEPXx2, TelXqx2 [16]	PB-N, CEPXx2, TelXqx2 [8]	no
19	28	6AA	NGS	seq(4)x1	-	PB-N, 4p11-q11x2 [16]	PB-N, 4p11-q11x2 [11]	no
20	41	6AA	NGS	seq(14)x1~2, (19)x2~3	-	PB-N, 14x2, 19x2 [11]	PB-N, 14x2, 19x2 [11]	no
21	41	6AA	NGS	mos seq(4p16.3p15.2)x3/(1-22)x2, (X,Y)x1	arr(1-22)x2, (X,Y)x1	PB-N, 4p16x2 [9]	PB-N, 4p16x2 [9]	no
22	41	4AA	NGS	mos seq(22)x1/ (1-22, X)x2	-	PB-N [9]	PB-N [11]	no
23	44	6AA	NGS	seq(8)x3	-	PB-N, 8x2 [19]	PB-N, 8x2 [22]	no

## Data Availability

The data presented in this study are available on request from the corresponding author.
